# Time-resolved serial femtosecond crystallography on fatty-acid photodecarboxylase: lessons learned

**DOI:** 10.1107/S2059798322007525

**Published:** 2022-08-25

**Authors:** Kyprianos Hadjidemetriou, Nicolas Coquelle, Thomas R. M. Barends, Elke De Zitter, Ilme Schlichting, Jacques-Philippe Colletier, Martin Weik

**Affiliations:** a Université Grenoble Alpes, CEA, CNRS, Institut de Biologie Structurale, 38000 Grenoble, France; b Max-Planck-Institut für medizinische Forschung, Jahnstrasse 29, 69120 Heidelberg, Germany; Stanford University, USA

**Keywords:** photoenzymes, time-resolved crystallography, structure-factor extrapolation, serial femtosecond crystallography, fatty-acid photodecarboxylase

## Abstract

Upon absorption of a blue-light photon, fatty-acid photodecarboxylase catalyzes the decarboxylation of free fatty acids to form hydrocarbons (for example alkanes or alkenes). The major components of the catalytic mechanism have recently been elucidated by combining static and time-resolved serial femtosecond crystallography (TR-SFX), time-resolved vibrational and electronic spectroscopies, quantum-chemical calculations and site-directed mutagenesis [Sorigué *et al.* (2021 ▸), *Science*, **372**, eabd5687]. The TR-SFX experiments, which were carried out at four different picosecond to microsecond pump–probe delays, yielded input for the calculation of Fourier difference maps that demonstrated light-induced decarboxylation. Here, some of the difficulties encountered during the experiment as well as during data processing are highlighted, in particular regarding space-group assignment, a pump-laser power titration is described and data analysis is extended by structure-factor extrapolation of the TR-SFX data. Structure refinement against extrapolated structure factors reveals a reorientation of the generated hydrocarbon and the formation of a photoproduct close to Cys432 and Arg451. Identification of its chemical nature, CO_2_ or bicarbonate, was not possible because of the limited data quality, which was assigned to specificities of the crystalline system. Further TR-SFX experiments on a different crystal form are required to identify the photoproducts and their movements during the catalytic cycle.

## Abstract 2

The crystallographic difficulties encountered in the data processing of recently published time-resolved serial femtosecond crystallography data are described. The origin of these issues is explained together with how they were circumvented or dealt with. The previously published crystallographic analyses are extended by the application of extrapolation methods to determine the structures of intermediate states.

## 1. Introduction

Time-resolved serial femtosecond crystallography (SFX; Barends *et al.*, 2022 ▸) at X-ray free-electron lasers (XFELs) presents a powerful means to study structural changes in crystalline biological macromolecules following reaction triggering (Colletier *et al.*, 2018 ▸; Brändén & Neutze, 2021 ▸). Because their activity can conveniently be triggered with light pulses, mostly photosensitive proteins have been studied by time-resolved SFX (TR-SFX) to date (Domratcheva & Schlichting, 2018 ▸; Poddar *et al.*, 2022 ▸), including myoglobin (Barends *et al.*, 2015 ▸), photoactive yellow protein (Pande *et al.*, 2016 ▸), photosystem II (Suga *et al.*, 2019 ▸; Kern *et al.*, 2018 ▸), various rhodopsins (Nango *et al.*, 2016 ▸; Nass Kovacs *et al.*, 2019 ▸; Skopintsev *et al.*, 2020 ▸; Oda *et al.*, 2021 ▸; Yun *et al.*, 2021 ▸; Mous *et al.*, 2022 ▸), phytochromes (Claesson *et al.*, 2020 ▸), a photosynthetic reaction center (Dods *et al.*, 2021 ▸), photoswitchable fluorescent proteins (Coquelle *et al.*, 2018 ▸), cytochrome *c* oxidase (Shimada *et al.*, 2017 ▸) and photolyase (Maestre-Reyna *et al.*, 2022 ▸), as well as P450nor (Tosha *et al.*, 2017 ▸; Nomura *et al.*, 2021 ▸) using a caged substrate. Most recently, TR-SFX has complemented other experimental and computational approaches in the study of the catalytic mechanism of fatty-acid photodecarboxylase (Sorigué *et al.*, 2021 ▸).

**Figure 1:**
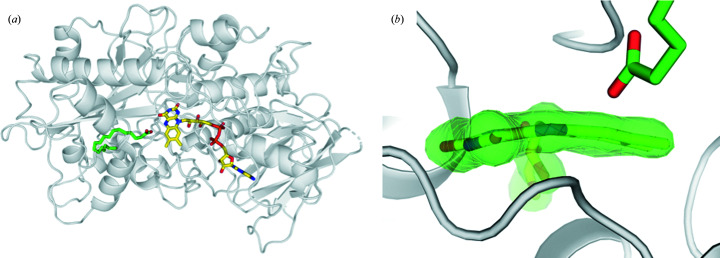
SFX dark-state crystal structure of *Cv*FAP (PDB entry 6zh7). (*a*) Structure of *Cv*FAP determined at 2 Å resolution from SFX data at room temperature (Sorigué *et al.*, 2021 ▸). (*b*) FAD is in a bent conformation (the C4—N5—N10—C9 dihedral angle is 14°). The *mF*_obs_ − *DF*_calc_ omit map at 3 r.m.s.d. (green) is overlaid. FAD and the C18 fatty-acid substrate are shown in yellow and green, respectively. The protein of monomer *A* is shown in gray.

Fatty-acid photodecarboxylase (FAP), together with protochlorophyllide oxidoreductase (Gabruk & Mysliwa-Kurdziel, 2015 ▸) and DNA photolyases (Sancar, 2016 ▸), is a member of the rare class of photoenzymes that require light to initiate each catalytic event. Absorption of a blue-light photon by the flavin adenine dinucleotide (FAD) cofactor within FAP triggers the decarboxylation of the fatty-acid substrate, which leads to the formation of a hydrocarbon molecule and CO_2_ (Sorigué *et al.*, 2017 ▸). The recently published high-resolution (1.8 Å) structure of FAP, determined from cryo-crystallo­graphic synchrotron data (see Fig. 1 in Sorigué *et al.*, 2021 ▸) combined with UV–Vis absorbance spectra, revealed a bent oxidized FAD in the dark state. A radiation damage-free dark-state SFX structure (Fig. 1 ▸) confirmed the bent nature of the FAD to be an unusual feature of the enzyme rather than the result of X-ray irradiation (Sorigué *et al.*, 2021 ▸). In this study, mechanistic insight into the photocatalysis of FAP was also obtained by combining experimental and computational approaches. Forward electron transfer from the fatty-acid substrate to the photoexcited FAD occurs in 300 ps and is accompanied by concomitant decarboxylation of the latter, as shown by time-resolved visible and infrared (IR) absorption spectroscopies, respectively. Back electron transfer from the 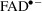 radical to the alkyl radical occurs in 100 ns, con­comitant with transformation of the generated CO_2_ into another molecule, possibly bicarbonate as suggested by FTIR. 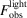 − 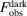 Fourier difference maps calculated from TR-SFX data at pump–probe delays of 20 ps, 900 ps, 300 ns and 2 µs indicated that decarboxylation had occurred by the 900 ps time point, in line with the rate determined by time-resolved IR (TR-IR) spectroscopy. Intermediate-state structures, however, were not presented in this study.

Here, we summarize the data-processing challenges that were encountered during the TR-SFX data analysis of Sorigué *et al.* (2021 ▸) and their possible origins. We present additional TR-SFX data collected at a pump–probe delay of 900 ps at three different pump-laser power densities that explain why the particular pump-laser power density was chosen for the TR-SFX experiment reported by Sorigué *et al.* (2021 ▸). Furthermore, structure-factor extrapolation was carried out for the four time points and controls that assess the quality of the resulting electron-density maps. Intermediate-state structures were then refined against extrapolated structure factors for the four time points. At 300 ns, the structure displays a repositioning of the hydrocarbon product with respect to the substrate. Different models were tested with the aim of identifying the compound that might explain the difference density seen in the active site at 300 ns.

## 2. Materials and methods

### 2.1. Brief summary of the previously reported TR-SFX data collection and processing (Sorigué *et al.*, 2021 ▸)

Needle-shaped microcrystals (10 × 4 × 4 µm ) of *Chlorella variabilis* FAP (*Cv*FAP) in 19%(*w*/*v*) PEG 4000, 0.1 *M* sodium citrate pH 5.5, 10 m*M* spermidine were injected with a gas dynamic virtual nozzle (GDVN; DePonte *et al.*, 2008 ▸) into the microfocus chamber of the CXI end station of the Linac Coherent Light Source (LCLS; Liang *et al.*, 2015 ▸) in November 2018 (proposal LT59). Optical pump [400 nm wavelength; circularly polarized; 4 ps (FWHM) pulse length; 11 µJ per pulse; 155 µm (FWHM) focal spot]–X-ray probe [9.5 keV photon energy; 23 fs pulse length; 1 µm (FWHM)] TR-SFX data were collected at 20 ps, 900 ps, 300 ns and 2 µs time delays. *CrystFEL* version 0.8.0 was used for indexing (*Xgandalf*; Gevorkov *et al.*, 2019 ▸), integration (*rings-grad* option) and merging with the Monte Carlo (MC) algorithm *process_hkl* (with scaling option). The high-resolution cutoff was 2 Å for all data sets, except for the 2 µs light data set, which was set at 2.2 Å. The space group is *P*2_1_, with two molecules (*A* and *B*) in the asymmetric unit and unit-cell parameters *a* = 61.4, *b* = 60.0, *c* = 182.9 Å, α = 90, β = 90.6, γ = 90° (Supplementary Table S1).

### 2.2. Pump-power titration at 900 ps

**Figure 2:**
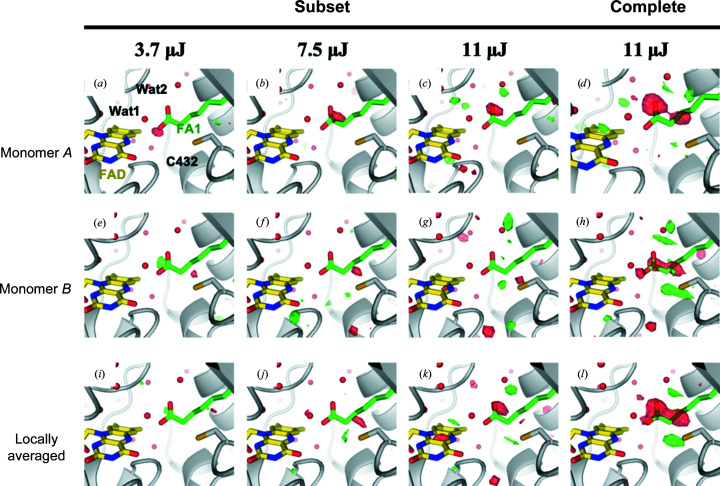
Fourier difference maps at 900 ps and three different pump-pulse energies using data processed in space group *P*2_1_. *q*-weighted Fourier difference electron-density maps calculated between SFX light (Δ*t* = 900 ps) data sets at different pump-laser energies and the dark data set ( 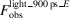 − 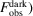 with *E* = 3.7 µJ (*a*, *e*, *i*), 7.5 µJ (*b*, *f*, *j*) and 11 µJ (*c*, *d*, *g*, *h*, *k*, *l*) at 2.2 Å resolution. Maps are contoured at +3.5 r.m.s.d. (green) and −3.5 r.m.s.d. (red). The SFX dark-state model (PDB entry 6zh7) of monomer *A* is overlaid in (*a*)–(*d*) and that of monomer *B* in (*e*)–(*l*), with FAD in yellow, the fatty-acid substrate in green and the protein in light gray. The maps were calculated with 68 421 dark images and with 18 704 light images in (*a*), (*c*), (*e*), (*f*), (*i*) and (*k*) (subset) and 50 214 light images in (*d*), (*h*) and (*l*) (complete).

At the start of the previously reported TR-SFX (LT59) experiment (Sorigué *et al.*, 2021 ▸), a limited pump-power titration was carried out at a 900 ps pump–probe delay using 7.5 µJ per pulse (nominally 1.9 absorbed photons per FAD when assuming similar absorption cross-sections for the first and subsequently absorbed photons), 3.7 µJ per pulse (nominally 0.9 photons per FAD) and 11 µJ per pulse (nominally 2.8 photons per FAD). The light data collected with pump-laser excitation at 3.7, 7.5 and 11 µJ per pulse consisted of 18 704, 34 264 and 50 214 indexed images, respectively, when processed in space group *P*2_1_ (Supplementary Table S2). The decision to carry out the subsequent TR-SFX series at 11 µJ per pulse was taken during the LT59 experiment based on *q*-weighted (Ursby & Bourgeois, 1997 ▸) Fourier difference electron-density maps 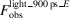 − 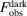 (Supplementary Fig. S1) using 18 430 dark images and 15 574, 12 796 and 19 151 light images available at the time (at pump energy values *E* of 3.7, 7.5 and 11 µJ per pulse, respectively) processed in space group *P*2_1_2_1_2_1_ (Supplementary Table S3) as assumed during the early phase of LT59 (see Section 3.1 for a detailed discussed of why the space group was initially assumed to be *P*2_1_2_1_2_1_ and was eventually chosen to be *P*2_1_). After completion of the LT59 experiment, the same number (18 704) of indexed images were randomly selected from the three light data sets processed in the correct *P*2_1_ space group (Supplementary Table S2) and 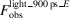 − 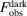 maps (Fig. 2 ▸) were calculated with *Xtrapol*8 (De Zitter *et al.*, 2022 ▸) using the 68 421 dark images published earlier (Sorigué *et al.*, 2021 ▸). Since certain parts of monomers *A* and *B* display significant conformational differences (Supplementary Fig. S2), these maps were averaged using a local averaging procedure (Nass *et al.*, 2020 ▸).

### 2.3. Calculation of Fourier difference electron-density maps and structure-factor extrapolation at four pump–probe delays

**Figure 3:**
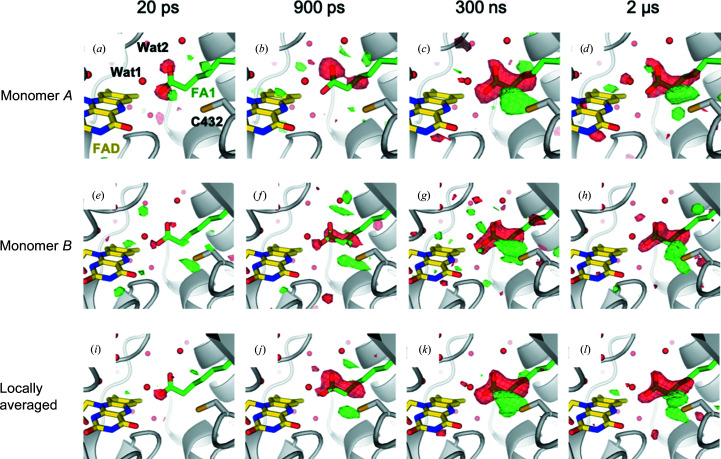
Fourier difference maps at four different pump–probe time delays. *q*-weighted Fourier difference electron-density maps calculated between the light and dark data sets ( 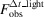 − 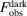 ) with Δ*t* = 20 ps (*a*, *e*, *i*), 900 ps (*b*, *f*, *j*) and 300 ns (*c*, *g*, *k*) at 2 Å resolution and Δ*t* = 2 µs (*d*, *h*, *l*) at 2.2 Å resolution. Maps corresponding to monomers *A* (*a*–*d*) and *B* (*e*–*h*) are shown at +3.5 r.m.s.d. (green) and −3.5 r.m.s.d. (red) and locally averaged maps (*i*–*l*) are shown at +4.0 r.m.s.d. (green) and −4.0 r.m.s.d. (red). The SFX dark-state model (PDB entry 6zh7) of monomer *A* is overlaid in panels *A*–*D* and of monomer *B* in panels *E*–*L*, with FAD in yellow, the fatty acid substrate in green and the protein in light gray.

*q*-weighted Fourier difference electron-density maps 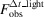 − 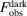 were calculated with *Xtrapol*8 using the dark-state structure (including the two fatty-acid substrates in the active site and at the protein surface) to phase the maps. As expected, these maps (Fig. 3 ▸) are similar to those published earlier (Sorigué *et al.*, 2021 ▸). We also used *Xtrapol*8 to determine the occupancy of the light states and to calculate extrapolated structure-factor amplitudes (*F*_ext_) using the formula (Duan *et al.*, 2013 ▸; Coquelle *et al.*, 2018 ▸) 

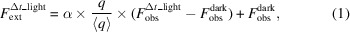

where α denotes the inverse of the occupancy, *q* and 〈*q*〉 are reflection-specific and average *q*-weights, and 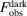 and 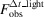 are the observed structure-factor amplitudes for the dark state and the photo-triggered state at a given time delay Δ*t*, respectively. The extrapolation procedure can generate negative *F*_ext_ that are not usable by refinement programs, resulting in reduced completeness (De Zitter *et al.*, 2022 ▸). The number of these reflections depends on the value of α and represents 1.58%, 3.50%, 6.02% and 2.41% of the extrapolated reflections in the data sets at Δ*t* = 20 ps, 900 ps, 300 ns and 2 µs, respectively, at the determined occupancies given below. To estimate their positive values, we used the *truncate* option in *Xtrapol*8, whereby a French–Wilson-based scaling is applied to all reflections (Evans & Murshudov, 2013 ▸). Occupancy determination was carried out using the *difference-map* method, which automates the procedure introduced in Coquelle *et al.* (2018 ▸) whereby the sum of the integrated values of selected peaks in the 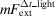 − 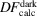 map is plotted as a function of the occupancy (Supplementary Fig. S3), and the occupancy value at the maximum peak height is considered to be correct. In the *difference-map* method, the highest peaks are automatically selected using a *Z* scoring of 2 on the normal distribution of all difference peaks in the *q*-weighted 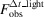 − 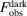 map, and are attributed to the closest residues to avoid possible bias that could skew occupancy determination. In the present case, the residues used for occupancy determination included Tyr466 and Cys432 and were all located around the fatty-acid substrate in the active site. The automatically determined occupancies lie within the range 25–35%. The maxima were observed at 35%, 30%, 25% and 35% for the 20 ps, 900 ps, 300 ns and 2 µs time delays, respectively, indicating the most probable sets of extrapolated structure-factor amplitudes were calculated.

**Figure 4:**
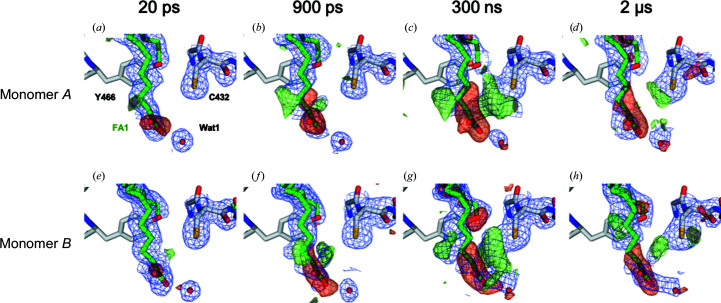
Extrapolated electron-density maps at four different pump–probe time delays calculated using the dark-state model. Extrapolated electron-density maps, 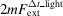 − 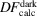 (1 r.m.s.d., blue) and 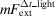 − 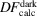 (+3 r.m.s.d., green; −3 r.m.s.d., red), calculated between the light and dark data sets with Δ*t* = 20 ps (*a*, *e*), 900 ps (*b*, *f*) and 300 ns (*c*, *g*) at 2 Å resolution and 2 µs (*d*, *h*) at 2.2 Å resolution. Maps are shown around the fatty acid (FA) and Cys432 of monomer *A* (*a*, *b*, *c*, *d*) and monomer *B* (*e*, *f*, *g*, *h*) and were calculated with the dark structure (including the two fatty-acid substrates and Wat1) as a phase model without refinement. The dark-state model is represented as sticks, with the C atoms of the protein in gray and those of the fatty-acid molecule in light green.

Difference density maps using the extrapolated structure factors ( 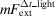 − 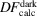 ; Fig. 4 ▸), were calculated using the dark-state structure (including the two fatty-acid substrates) as a phase model. Both 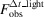 − 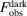 (Fig. 3 ▸) and 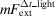 − 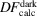 (Fig. 4 ▸) maps indicate structural changes that occur at time delay Δ*t* with respect to the dark state.

### 2.4. Difference refinement using extrapolated structure factors

In order to model the structural changes that had occurred at Δ*t*, difference refinement of the Δ*t*_light structures was performed against 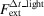 using *phenix.refine* (Afonine *et al.*, 2012 ▸) from the *Phenix* suite (Liebschner *et al.*, 2019 ▸). The refinement started from the dark model (PDB entry 6zh7), in which the two fatty-acid substrates were omitted and the atom coordinates were randomized with a mean error value of 0.5 Å using *phenix.pdbtools*. Positional and isotropic individual *B*-factor refinement was carried out in reciprocal space using wxc_scale = 0.02 and secondary-structure restraints as required for maximum-likelihood refinement to converge. Simulated annealing was performed during the first cycle of refinement using the default parameters of *phenix.refine*. Manual model building and real-space refinement were performed using *Coot* (Emsley *et al.*, 2010 ▸).

**Figure 5:**
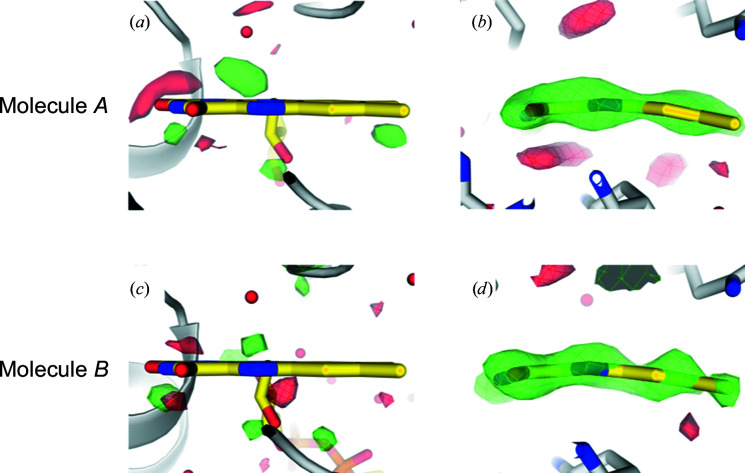
Conformation of the isoalloxazine ring of the FAD cofactor in the extrapolated structure at 300 ns. Extrapolated electron-density maps, 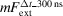 − *DF*_calc_ (+3 r.m.s.d., green; −3 r.m.s.d., red), calculated between the dark and the light data set at 300 ns and phased with a model in which the isoalloxazine rings of the FAD cofactor (yellow) are either restrained to be planar (*a*, *c*) or absent (*b*, *d*). In (*a*) and (*c*) a model with a planar FAD is superimposed and in (*b*) and (*d*) the final refined light model at 300 ns is superimposed, in which the isoalloxazine bending angle is ∼10°.

Particular attention was paid to modeling the FAD cofactor. When its isoalloxazine rings were forced to be planar or were omitted from the model at 300 ns, the 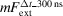 − *DF*_calc_ map displayed peaks indicative of FAD bending (Fig. 5 ▸). In the final refined light model at 300 ns, the isoalloxazine ring system deviates from planarity by ∼10° (C4—N5—N10—C9 dihedral angle; Figs. 5 ▸ *b* and 5 ▸ *d*). Similarly, the deviation from planarity is 11°, 9° and 10° in the refined light models at 20 ps, 900 ps and 2 µs, respectively. The corresponding angle in the SFX dark-state structure was determined to be 14° (Sorigué *et al.*, 2021 ▸).

Before modeling the electron density with potential reaction products, the quality of the extrapolated electron-density maps was assessed by omitting a well ordered water molecule (Wat2) and the rigid active-site side chains of Arg451 and Trp479 from the model at 300 ns and calculating 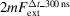 − *DF*_calc_ and 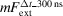 − *DF*_calc_ maps (Supplementary Fig. S4). Since electron density for both side chains and for Wat2 was present, modeling of the reaction products was attempted.

**Figure 6:**
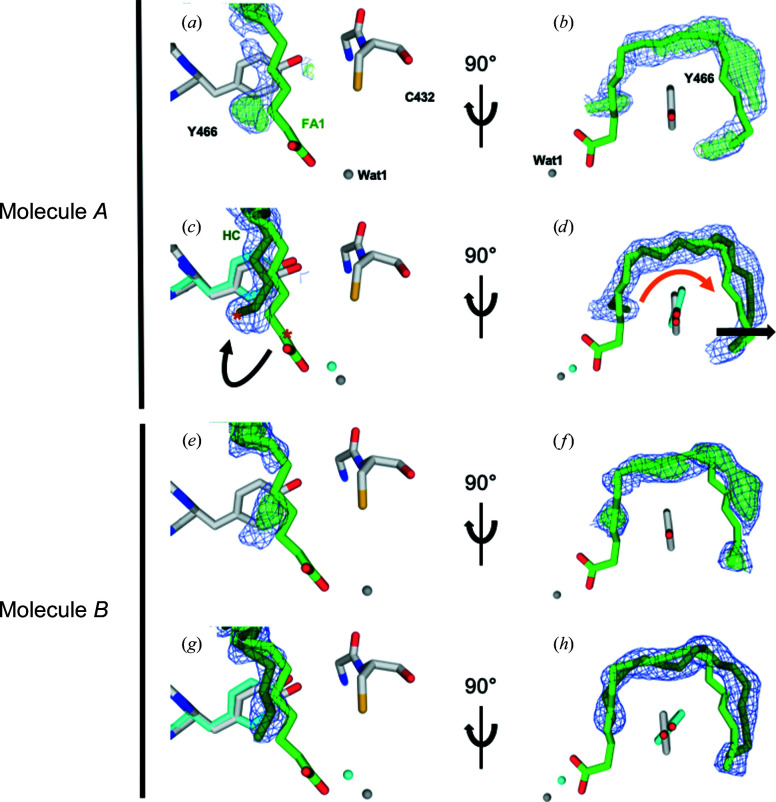
Hydrocarbon product in the extrapolated structure at 300 ns. (*a*, *b*, *e*, *f*) *q*-weighted extrapolated electron-density maps, 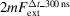 − *DF*_calc_ (1 r.m.s.d., blue mesh) and 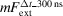 − *DF*_calc_ (+3 r.m.s.d., green), calculated with models of monomers *A* (*a*, *b*) and *B* (*e*, *f*) from which the substrate was omitted. (*c*, *d*, *g*, *h*) Extrapolated 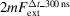 − *DF*_calc_ electron-density maps (1 r.m.s.d., blue mesh) calculated with models of monomers *A* (*c*, *d*) and *B* (*g*, *h*) without substrate but including a hydrocarbon product (HC; dark green). Dark-state and 300 ns intermediate-state models of monomer *A* (*a*, *b*, *c*, *d*) and monomer *B* (*e*, *f*, *g*, *h*) are overlaid in gray and cyan, respectively. The fatty-acid substrate (FA1) in the dark model is shown in lime green in (*a*)–(*h*)

**Figure 7:**
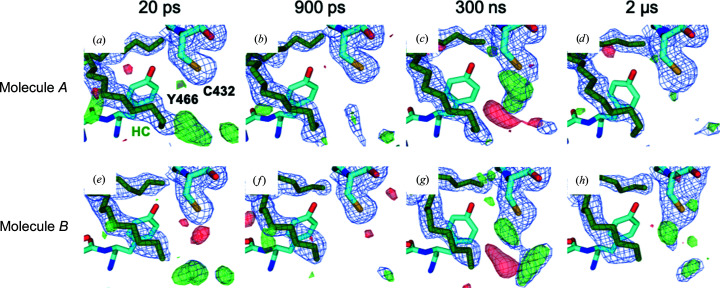
Extrapolated electron-density maps at four different pump–probe time delays calculated using refined models containing a C17 hydrocarbon molecule. Extrapolated electron-density maps, 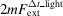 − 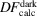 (1 r.m.s.d., blue) and 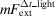 − 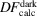 (+3 r.m.s.d., green; −3 r.m.s.d., red), calculated between the light and dark data sets with Δ*t* = 20 ps (*a*, *e*), 900 ps (*b*, *f*) and 300 ns (*c*, *g*) at 2 Å resolution and 2 µs (*d*, *h*) at 2.2 Å resolution. Maps are shown around the fatty acid (FA) and Cys432 of monomer *A* (*a*, *b*, *c*, *d*) and monomer *B* (*e*, *f*, *g*, *h*) and were calculated with refined models containing a C17 hydrocarbon molecule. The respective refined models are represented as sticks, with the C atoms of the protein in cyan and the hydrocarbon in dark green. Note that Wat1 has been excluded from all models.

At first, the focus was on modeling the alkane product. We outline the approach again using the 300 ns data as an example. 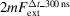 − *DF*_calc_ and 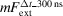 − *DF*_calc_ maps calculated either with the dark-state model (PDB entry 6zh7; Figs. 4 ▸ *c* and 4 ▸ *g*) or with a model from which the substrate had been omitted (Figs. 6 ▸ *a*, 6 ▸ *b*, 6 ▸ *e* and 6 ▸ *f*) suggested that a C17 hydrocarbon molecule should be modeled (Figs. 6 ▸ *c*, 6 ▸ *d*, 6 ▸ *g* and 6 ▸ *h*). Similarly, a C17 hydrocarbon molecule was modeled at the other three time points (Fig. 7 ▸; Supplementary Table S4).

**Figure 8:**
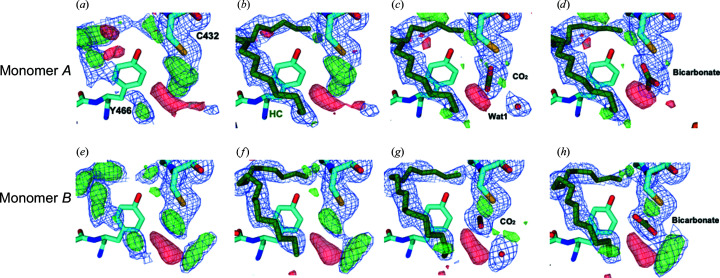
Attempts to model various molecules next to Cys432 at 300 ns. Extrapolated electron-density maps, 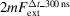 − *DF*_calc_ (1 r.m.s.d., blue mesh) and 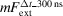 − *DF*_calc_ (+3 r.m.s.d., green; −3 r.m.s.d., red), calculated between the dark and light data sets at 300 ns and phased with a model without (*a*, *e*) and with (*b*, *f*) the hydrocarbon molecule (HC), but without any additional molecule next to Cys432, with a CO_2_ and water molecule both at 100% occupancy (*c,* *g*) or with an 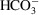 molecule at 100% occupancy (*d*, *h*). The corresponding models of monomers *A* (*a*, *b*, *c*, *d*) and *B* (*e*, *f*, *g*, *h*) are shown. 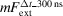 − *DF*_calc_ omit maps for Wat1 are shown in (*b*) and (*f*).

Before and after modeling the hydrocarbon molecule at 300 ns, there is a strong positive feature next to the side chain of Cys432 in the 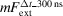 map in both monomers *A* (Figs. 8 ▸ *a* and 8 ▸ *b*) and *B* (Figs. 8 ▸ *e* and 8 ▸ *f*) at a similar position to a positive peak seen in the 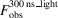 – 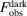 maps (Figs. 3 ▸ *c*, 3 ▸ *g* and 3 ▸ *k*). Two different models were assessed to fit this positive peak: a CO_2_ and a water molecule, both at 100% occupancy (Figs. 8 ▸ *c* and 8 ▸ *g*), or an 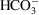 molecule at 100% occupancy (Figs. 8 ▸ *d* and 8 ▸ *h*). The correlation between the models of monomers *A* and *B* (including either CO_2_ and a water or 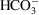 ) and the corresponding map was calculated with *phenix.get_cc_mtz_pdb* using the scale option and fixing a 3 Å radius around the atoms of the products.

## 3. Results and discussion

### 3.1. Choice of space group, twinning and data quality

Prior to the TR-SFX experiment described here (LCLS, LT59, November 2018), needle-shaped *Cv*FAP microcrystals were used in a short test run at the LCLS (LR38; February 2018). The space group was found to be *P*2_1_2_1_2_1_, with unit-cell parameters *a* = 60, *b* = 70, *c* = 115 Å. During the scale-up phase in preparation for LT59, the imidazole/maleate buffer was replaced by sodium citrate, with all other crystallization parameters remaining unchanged. This replacement allowed the needle-shaped crystals to grow thicker. Due to time restrictions, the crystals could not be tested at a synchrotron prior to experiment LT59, at the beginning of which we thus assumed the space group to be *P*2_1_2_1_2_1_. We could indeed index the diffraction patterns according to an orthorhombic lattice type; however, the unit-cell parameters *a* = 61, *b* = 60, *c* = 180 Å indicated a change in crystal form. The observation of two populations of α angles, distributed sharply around 89.3° and 90.5° (Supplementary Fig. S5*a* ), led us to re-index all data according to a monoclinic lattice and merging intensities specifying *P*2_1_, using unit-cell parameters *a* = 61.4, *b* = 60.0, *c* = 182.9 Å, α = 90.0, β = 90.6, γ = 90.0° (Supplementary Fig. S5*b* ).

The indexing nonetheless remained ambiguous, as can be judged from the relatively high *R*_split_ values reported in Sorigué *et al.* (2021 ▸) for the dark data set (15.1% and 68.5% for the overall *R*_split_ and in the highest resolution shell, respectively; Supplementary Table S1). Indeed, the lattice displayed higher point-group symmetry, *mmm*, than expected for space group *P*2_1_, which would be 2/*m*. Because *a* ≃ *b* ≃ *c*/3, an indexing ambiguity can arise from swapping, for example, the *a* and *b* axes or cyclic permutation of the axes. However, if this had happened during indexing or by actual twinning, the former would lead to a peak in the 90° section of the self-rotation function and the latter to a peak in the 120° section, neither of which is observed (not shown). Nevertheless, a small fraction of misindexed patterns would not generate a peak in the self-rotation function but would still affect the intensity statistics. The only remaining possibility for twinning (or misindexing) is a 180° rotation around *a* or *c*, which is possible because β is close to 90°. Due to the crystallographic twofold axis, a rotation of 180° around *a* or *c* is nearly equivalent and would manifest as peaks in the 180° section of the self-rotation function. These are indeed observed and are of approximately the same height as the crystallographic peak (Supplementary Fig. S6; calculated using *MOLREP* from the *CCP*4 suite; Winn *et al.*, 2011 ▸), which could indicate ∼50% twinning. Based on the *L*-test (Padilla & Yeates, 2003 ▸), however, twinning could be excluded. However, there is also noncrystallographic symmetry (NCS) relating the two monomers *A* and *B* in the asymmetric unit of the monoclinic space group, which is a twofold rotation (almost) perpendicular to the crystallographic twofold axis, which results in the creation of a third twofold perpendicular to the other two. Thus, the contents of the unit cell indeed have approximate *mmm* point group symmetry, and even without twinning the strong peaks in the 180° section of the self-rotation function are expected. Accordingly, the *P*2_1_ packing is only a minor deviation from the *P*2_1_2_1_2_1_ packing (Supplementary Fig. S9).

**Figure 9:**
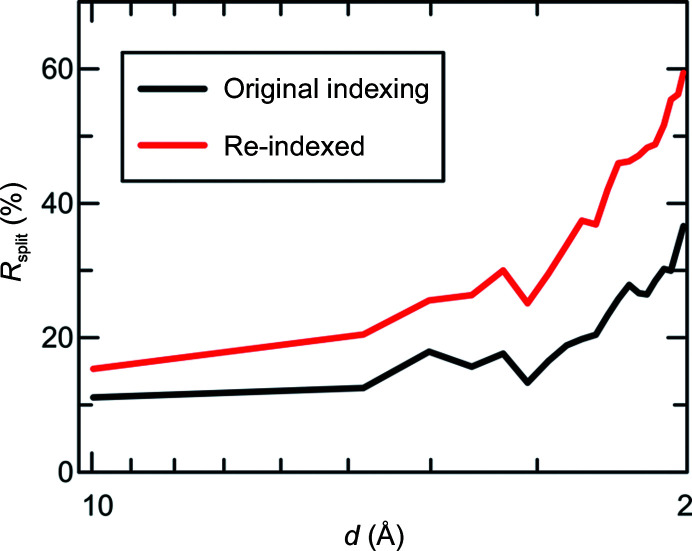
Comparison of the *R*-factor intensity distribution between native and re-indexed data sets. *R*_split_ as a function of resolution for the *Cv*FAP dark data before (black line) and after (red line) re-indexing one of the two half data sets using the operation *h*, −*k*, −*l*. Applying this operation, which is a member of space group *P*2_1_2_1_2_1_, results in a noticeable increase in *R*_split_, suggesting that the true symmetry is *P*2_1_.

To further investigate whether the symmetry is *P*2_1_ or *P*2_1_2_1_2_1_, we split the dark images into two equal halves that were integrated separately using *P*2_1_ space-group symmetry and calculated *R*_split_, *i.e.* the *R* factor between the two sets of intensities derived from the two half data sets corrected for the decrease in the number of observations caused by dividing the data into halves (White *et al.*, 2012 ▸). We then applied the re-indexing operator *h*, −*k*, −*l* (one of the symmetry operations of *P*2_1_2_1_2_1_) to one of the two half data sets and again calculated *R*_split_. This procedure, proposed by an anonymous referee, allows the two possible space-group choices to be compared on the basis of *R*_split_ values calculated using the same number of reflections, which would not be the case when comparing *R*_split_ values obtained from processing all the data in either *P*2_1_ or *P*2_1_2_1_2_1_. In this case, re-indexing one of the two half data sets resulted in much higher values of *R*_split_, particularly at high resolution (Fig. 9 ▸). Thus, any *h*, −*k*, −*l* symmetry in the data is not perfect, and the true space-group symmetry of the data is therefore most likely to be *P*2_1_.

As an alternative to merging intensities by Monte Carlo (MC) averaging (Sorigué *et al.*, 2021 ▸), merging with *partialator* (--custom-split option) was carried out in *CrystFEL* version 0.8.0, which resulted in a decreased *R*_split_ of the dark data set of 12.1% (15.1% for MC), but yielded apparently twinned data as assessed with *phenix.xtriage* (not shown). Also, up to ∼22% of the measured reflections were discarded by *partialator*, suggesting that the gain in precision of the data could lead to reduced accuracy in the estimation of structure-factor amplitudes. Therefore, we decided to rely on the merged intensities obtained by MC averaging. Nevertheless, we cannot exclude that the use of *partialator* yielded data that were so much better that real twinning could be detected.

After completion of the LT59 beamtime, diffraction data were collected from single cryo-cooled *Cv*FAP crystals on beamline PXII-X10SA at SLS. The space group varied between *P*2_1_2_1_2_1_ and *P*2_1_ from crystal to crystal, sometimes even as a function of the data-acquisition location on the long needle-shaped crystals.

In summary, the relatively large *R*_split_ values (see Supplementary Table S1, which reproduces Table S2 from Sorigué *et al.*, 2021 ▸) are likely to reflect inherent variability in the data that could stem from indexing ambiguities.

### 3.2. Effect of pump-laser energy on Fourier difference maps at 900 ps

The appropriate optical pump-laser energy to use in a TR-SFX experiment is currently a much-debated issue. Motivated by the wish to increase the magnitude of light-induced features, all studies have been carried out so far at laser energies that can result in one or more absorbed photon/chromophore, carrying the risk of unwanted multiphoton effects contaminating or even dominating the functionally relevant single-photon processes (Grünbein *et al.*, 2020 ▸; Miller *et al.*, 2020 ▸). Good practice is thus to carry out a spectroscopic pump-laser power titration on protein crystals or solutions to identify the linear excitation regime (see, for example, Hutchison *et al.*, 2016 ▸; Nass Kovacs *et al.*, 2019 ▸; Sorigué *et al.*, 2021 ▸), ideally followed by a structural power titration to assess whether structural changes can be seen in that regime (see, for example, Claesson *et al.*, 2020 ▸).

Our recent TR-SFX study presented structural data at four pump–probe delays (20 ps, 900 ps, 300 ns and 2 µs) acquired after a pump pulse of 11 µJ, an energy corresponding to an average of 2.8 nominally absorbed photons per chromophore (Sorigué *et al.*, 2021 ▸). Prior to collecting data at the four time points using this laser energy, a limited number of light images were collected at 900 ps with 3.7 and 7.5 µJ per pulse (0.9 and 1.9 nominally absorbed photons per chromophore per pulse on average, respectively). 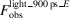 − 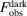 Fourier difference maps were calculated between the light and dark data merged in *P*2_1_2_1_2_1_, *i.e.* in the space group that we assumed at the beginning of the LT59 beamtime (Supplementary Fig. S1), based on 18 430 dark images and 15 574, 12 796 and 19 151 light images at 3.7, 7.5 and 11 µJ per pulse, respectively. Negative peaks on the fatty-acid carboxyl group are present, the height of which increases as a function of the laser energy. This increase motivated our choice of collecting the subsequent TR-SFX data at 11 µJ per pulse. A better-informed decision could have been made if we had integrated the negative difference electron around the fatty-acid carboxyl group and plotted the values as a function of pump-laser energy to check whether the signal increases linearly with the pump energy.

After completion of the LT59 beamtime, 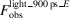 − 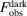 maps were calculated again based on data merged in *P*2_1_, *i.e.* in the space group that we eventually considered to be more likely than *P*2_1_2_1_2_1_ (Fig. 2 ▸; Supplementary Table S2). The maps were calculated with 68 421 dark images and with an equal number of 18 704 light images for the three 900 ps light data sets, which correspond to a subset of the available 7.5 and 11 µJ per pulse data, respectively. At the three pump energies negative difference density peaks are observed at different atoms of the fatty-acid carboxylate for monomer *A* (Figs. 2 ▸ *a*–2 ▸ *c*); for monomer *B* no peaks are observed (Figs. 2 ▸ *e*–2 ▸ *g*). This is not due to a lack of photocleavage since strong negative peaks are observed in both monomers, covering almost the entire carboxylate, when almost three times as many light images (*i.e.* 50 214) are used for map calculation at 11 µJ (Fig. 2 ▸ *h*). Together, this clearly shows that the data derived from the 18 704 images collected for the power titration are not accurate enough to assess the extent of photolysis with confidence. Many more images should have been collected (for a discussion of the signal-to-noise ratio as a function of indexed images, see Gorel *et al.*, 2021 ▸).

### 3.3. Fourier difference maps, structure-factor extrapolation and intermediate-state models

Fourier difference density maps calculated between light and dark data sets mainly show peaks in the active site (see, as an example, the 300 ns map covering an entire asymmetric unit; Supplementary Fig. S7) and provide clear evidence for substrate decarboxylation (Fig. 5 in Sorigué *et al.*, 2021 ▸). Here, Fourier difference electron-density maps with a very similar content were reproduced with *Xtrapol*8 (De Zitter *et al.*, 2022 ▸) (Fig. 3 ▸). Two observations are noteworthy. Firstly, the apparent extent of decarboxylation seems to be different at 900 ps (Figs. 3 ▸ *b* and 3 ▸ *f*) and 300 ns (Fig. 3 ▸ *c* and 3 ▸ *g*), a surprising observation in view of the decarboxylation time constant of 270 ps determined by TR-IR spectroscopy (Sorigué *et al.*, 2021 ▸). Three possible explanations can be offered: (i) the spatial overlap between the pump and probe laser changed slightly, (ii) the apparent difference reflects the noise level of the data or (iii) at 900 ps a positive peak due to photodecarboxylated CO_2_ compensates part of the negative peak on the carboxyl group. Secondly, it is striking that the peaks behave differently, both in terms of height and temporal evolution, in monomers *A* and *B*. It is very likely that this reflects noise in the data since the photochemical decarboxylation yield is expected to be the same in both monomers; nevertheless, differences in protein dynamics may also be possible due to a different packing environment (Supplementary Fig. S8), which would be in line with the conformational differences identified at the protein surface (Supplementary Fig. S2).

In order to model the structural changes that had occurred at the different time points, structure-factor extrapolation was carried out using *Xtrapol*8 (see Section 2 and De Zitter *et al.*, 2022 ▸), which estimates the structure-factor amplitudes *F*_ext_ that would have been measured for each pump–probe data set if the photo-triggered intermediate were present in the crystals at 100% occupancy (equation 1). The occupancy of intermediate states was determined to be between 25% and 35% for the four time delays. Extrapolated 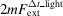 − 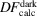 and 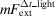 − 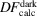 electron-density maps (Fig. 4 ▸) point to qualitatively similar structural changes as the Fourier difference electron-density maps (Fig. 3 ▸) for the four time delays. In particular, a negative 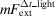 − 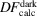 peak on the substrate reflects light-induced decarboxylation and a nearby positive peak indicates reorientation of the formed hydrocarbon chain (Fig. 4 ▸). At 300 ns and 2 µs a positive 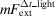 − 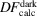 peak is visible next to the side chain of Cys432, in line with the reported observations in Fourier difference density maps (Sorigué *et al.*, 2021 ▸).

Before modeling reaction products, the information content of the extrapolated electron-density maps was evaluated by calculating omit maps. The 300 ns data serve as an example: the well ordered water molecule WAT2 and the side chains of the rigid active-site residues Arg451 and Trp479 were removed from the dark-state structure and the resulting model was used to phase electron-density maps using extrapolated structure factors (Supplementary Fig. S4). These maps ( 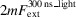 − *DF*_calc_ and 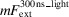 − *DF*_calc_) show clear electron density for the omitted atoms in both monomers, indicating that the extrapolated structure factors contain sufficient information to correctly locate large, rigid side chains and well ordered water molecules. Further, the conformation of the isoalloxazine ring of the FAD cofactor, which is bent in the dark-state structure (Sorigué *et al.*, 2021 ▸), was assessed at 300 ns by restraining it to be planar. The resulting 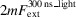 − *DF*_calc_ and 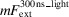 − *DF*_calc_ maps (Figs. 5 ▸ *a* and 5 ▸ *c*) indicated cofactor bending, which was determined to be 10° in the final refined 300 ns light-state structure (Figs. 5 ▸ *b* and 5 ▸ *d*).

To identify and locate reaction products, the initial focus was on the hydrocarbon product (C17), as again illustrated by the 300 ns data. Firstly, 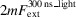 − *DF*_calc_ and 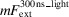 − *DF*_calc_ maps based on a model without fatty-acid substrate or hydrocarbon product were calculated (Figs. 6 ▸ *a*, 6 ▸ *b*, 6 ▸ *e* and 6 ▸ *f*). These indicate that the hydrocarbon moves towards the side chain of Tyr466 (Figs. 6 ▸ *a* and 6 ▸ *e*) and recoils (Figs. 6 ▸ *b* and 6 ▸ *f*) with respect to the fatty-acid position, as evident in the final model (Figs. 6 ▸ *c*, 6 ▸ *d*, 6 ▸ *g* and 6 ▸ *h*). Recoil of the hydrocarbon product is accompanied by a small rotation in the side chain of Tyr466 (Figs. 6 ▸ *d* and 6 ▸ *h*), as observed previously in a synchrotron cryo-crystallography structure (Sorigué *et al.*, 2021 ▸). A hydrocarbon chain was then included in the light models at the 20 ps, 900 ps and 2 µs time points and refined against extrapolated structure factors (Supplementary Table S4; Fig. 7 ▸). At 20 ps, but not at the other time points, the extrapolated electron-density maps indicated that a fatty-acid substrate rather than a hydrocarbon product needed to be modeled in the active site (Fig. 4 ▸), in line with the decarboxyl­ation time constant of 270 ps determined by TR-IR spectroscopy (Sorigué *et al.*, 2021 ▸).

No products other than an alkane molecule were modeled in the light structures at 20 and 900 ps because no residual peaks in the 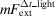 − 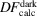 maps (Figs. 4 ▸ *a*, 4 ▸ *b*, 4 ▸ *e* and 4 ▸ *f*) were present that would have indicated the necessity of doing so. At 300 ns, however, further modeling was attempted to assess whether structure-factor extrapolation could help to decide between a bicarbonate ion and CO_2_, an ambiguity debated in Sorigué *et al.* (2021 ▸). Indeed, our earlier report suggested, but did not prove, the transient formation of a bicarbonate molecule next to Cys432 after decarboxylation (Sorigué *et al.*, 2021 ▸). In an attempt to test this suggestion, we either modeled a CO_2_ and a water molecule (Figs. 8 ▸ *c* and 8 ▸ *g*) or a bicarbonate molecule (Figs. 8 ▸ *d* and 8 ▸ *h*) at 300 ns and refined against extrapolated structure factors. We note that the bicarbonate position in monomer *B* (Fig. 8 ▸ *h*), but not in monomer *A* (Fig. 8 ▸ *d*), is similar to that suggested based on cryo-crystallographic data (Fig. 4*d* in Sorigué *et al.*, 2021 ▸). Peaks in the residual 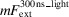 − *DF*_calc_ map do not allow one to clearly discriminate between these two models, and the 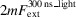 − *DF*_calc_ maps tend to support both to a similar level (Fig. 8 ▸). The *R*_work_ and *R*_free_ values of 34.9% and 40.8%, respectively, for the CO_2_/water model (model 1) and 34.4% and 40.9%, respectively, for the HCO_3_^−^ model (model 2) indicate a minor decrease of the *R*_free_ value by 0.1 for model 1. Real-space correlation between map coefficients and CO_2_ or 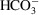 of monomers *A* and *B* in the corresponding structures indicates a slightly better correlation for model 2 (CC = 0.724 and 0.57 for monomers *A* and *B*, respectively) than for model 1 (CC = 0.717 and 0.51 for monomers *A* and *B*, respectively). Therefore, electron-density maps calculated from extrapolated structure factors do not allow one to resolve the product ambiguity at 300 ns. At 2 µs, further product modeling was not attempted because the peaks next to Cys432 were lower (Fig. 3 ▸ *d* and 3 ▸ *h*) than at 300 ns (Figs. 3 ▸ *c* and 3 ▸ *g*), possibly due to the limited data quality at 2 µs (Supplementary Tables S1 and S4).

## 4. Conclusions

We discuss several lessons that were learnt during the course of the recently reported TR-SFX study on *Cv*FAP (Sorigué *et al.*, 2021 ▸). Firstly, a minor change in the batch crystallization conditions (sodium citrate instead of imidazole/maleate) during the scale-up phase led to an unexpected change in the space group and the unit-cell parameters of the needled-shaped crystals. Assessing the diffraction of the final crystal batch at a synchrotron source prior to the TR-SFX experiment could have uncovered some of the problematic features of the needle-shaped *Cv*FAP microcrystals. Indeed, the peculiar unit-cell characteristics (*a* ≃ *b* ≃ *c*/3, β close to 90°) and the noncrystallographic symmetry axis being close to a crystallographic axis probably led to an indexing ambiguity that could not be solved and resulted in poor data statistics, such as high *R*_split_ values. Furthermore, we learnt that the mandatory pump-laser power titration needs to be based on a large enough number of images to yield high signal to noise in electron-density maps for the feature investigated (here decarboxylation; Gorel *et al.*, 2021 ▸) and on more than the three energies used here. If Fourier difference electron-density maps with high signal to noise had been available, peaks could have been integrated and plotted as a function of pump-laser energy in order to be able to choose conditions that were still in the linear excitation regime.

Here, we extend the study of Sorigué *et al.* (2021 ▸) by carrying out the refinement of intermediate-state structures against extrapolated structure factors at 20 ps, 900 ps, 300 ns and 2 µs. A particular focus was on the 300 ns light structure, which shows a reorientation of the hydrocarbon product after photodecarboxylation of the fatty-acid substrate and displays a FAD cofactor that is similarly bent to that in the dark-state structure. Refinement against extrapolated structure factors at 300 ns did not allow distinction between two possible products located near Cys432. This will require further TR-SFX studies on *Cv*FAP in a less problematic crystal form.

## Supplementary Material

PDB reference: difference-refined structure of fatty-acid photodecarboxylase following 400 nm laser irradiation of the dark state, after 20 ps, 7r33

PDB reference: after 900 ps, 7r34

PDB reference: after 300 ns, 7r35

PDB reference: after 2 µs, 7r36

Supplementary Tables and Figures. DOI: 10.1107/S2059798322007525/wa5138sup1.pdf
