# Supplementary material for: Time-resolved serial femtosecond crystallography on fatty-acid photodecarboxylase: lessons learned
Source: Acta Crystallogr D Struct Biol. 2022 Aug 25;78(Pt 9):1131–42. doi: 10.1107/S2059798322007525 (PMC9435596; doi:10.1107/S2059798322007525)
Supplement: Supplementary file 1 [file d-78-01131-sup1.pdf]

STRUCTURAL
BIOLOGY

Volume 78 (2022)

Supporting information for article:

Time-resolved serial femtosecond crystallography on fatty-acid photodecarboxylase: lessons learned

Kyprianos Hadjidemetriou, Nicolas Coquelle, Thomas R. M. Barends, Elke De Zitter, Ilme Schlichting, Jacques-Philippe Colletier and Martin Weik

Supporting information

Supplementary Table S1: Statistics of data processing and of refinement against observed structure factors at 20 ps, 900 ps, 300 ns, 2 µs as reported in Sorigué *et al* ((Sorigue *et al.*, 2021))

Dataset	dark	light_20ps	light_900ps	light_300ns	light_2µs
PDB ID code	6ZH7				
Pump-laser excitation (400 nm)	no	yes	yes	yes	yes
Pump energy (µJ)	n/a	11	11	11	11
Nominal pump-probe delay	n/a	20 ps	900 ps	300 ns	2 µs
Space group	$P2_1$	$P2_1$	$P2_1$	$P2_1$	$P2_1$
Unit cell parameters<br>a (Å)<br>b (Å)<br>c (Å)<br>β (°)	 61.4± 0.1<br>60.0 ± 0.1<br>182.9 ± 0.3<br>90.6	 61.4 ± 0.1<br>60.0 ± 0.1<br>182.9 ± 0.3<br>90.6	 61.4 ± 0.1<br>60.0 ± 0.1<br>182.9 ± 0.3<br>90.6	 61.4 ± 0.1<br>60.0 ± 0.1<br>182.9 ± 0.3<br>90.6	 61.4 ± 0.1<br>60.0 ± 0.1<br>182.9 ± 0.3<br>90.6
Collected frames	2,579,455	1,625,450	1,728,093	1,298,912	909,645
Hits	264,812	290,962	227,517	151,599	66,358
Indexed images	68,421	88,919	50,214	44,868	18,600
Resolution (Å)	25– 2.00 (2.05 – 2.00)	25– 2.00 (2.05 – 2.00)	25– 2.00 (2.05 –2.00)	25– 2.00 (2.05 – 2.00)	25– 2.20 (2.25 – 2.20)
Observations	33,069,955 (1,438,474)	42,928,992 (1,869,092)	25,083,092 (1,091,803)	20,934,706 (907,081)	8,076,033 (383,644)
Unique reflections	93,061 (6,086)	93,060 (6,086)	93,064 (6,086)	93,055 (6,086)	70,385 (4,671)
$R_{split}^{\#}$ (%)	15.1 (68.5)	13.4 (61.3)	18.0 (80.0)	19.4 (84.5)	24.9 (67.8)
CC*	0.996 (0.841)	0.996 (0.869)	0.994 (0.797)	0.992 (0.790)	0.983 (0.816)
I / σ(I)	5.6 (1.7)	6.40 (1.9)	4.8 (1.4)	4.4 (1.3)	3.7 (1.6)
Completeness (%)	100 (100)	100 (100)	100 (100)	100 (100)	100 (100)
Multiplicity	355 (236)	461 (307)	270 (179)	225 (149)	115 (82)
$R_{iso}^{\$}$ (with respect to dark dataset)	n.a	0.157	0.171	0.174	0.202
Refinement statistics					
Refinement strategy	Classical refinement	n.a	n.a	n.a	n.a
Resolution (Å)	25– 2.00 (2.05 – 2.00)				
R_{free}	0.235				
R_{work}	0.196				
Number of protein atoms	8417				
Number of ligand atoms	166				
Number of water atoms	394				

B-factor protein (Å^2)	31				
r.m.s.d. bond lengths (Å)	0.01				
r.m.s.d. angles (°)	1.8				
Ramachandran favored	95.8 %				
Ramachandran allowed	3.7 %				
Ramachadran outliers	0.2 %				
Rotamer outliers	1.6 %				
C-beta outliers	0				
Clashscore	5				

Values in brackets are for the highest resolution shell

Supplementary Table S2: Statistics of pump-power titration SFX data processed in space group *P*2_1

Dataset	Δt_900 ps	Δt_900 ps	Δt_900 ps
Energy (μJ)	3.7	7.5	11
Data collection and processing			
Space group	*P*2_1	*P*2_1	*P*2_1
$^{\$}$Unit cell parameters<br>a (Å)<br>b (Å)<br>c (Å)<br>β (°)	 <br>61.4 ± 0.1<br>60.0 ± 0.1<br>182.9 ± 0.3<br>90.6	 <br>61.4 ± 0.1<br>60.0 ± 0.1<br>182.9 ± 0.3<br>90.6	 <br>61.4 ± 0.1<br>60.0 ± 0.1<br>182.9 ± 0.3<br>90.6
Hits	85,757	141,349	188,288
Indexed image, total	18,704	34,264	50,214
Indexing rate (%)	21.81	24.24	26.67
Indexed images, subset	18,704	18,704	18,704 (*)
Resolution (Å)	25– 2.20<br>(2.25 – 2.20)	25– 2.20<br>(2.25 – 2.20)	25– 2.20<br>(2.25 – 2.20)
Observations	7,724,496<br>(365,950)	10,189,805<br>(490,193)	10,751,282<br>(515,733)
Unique reflections	70,419 (4,672)	70,420 (4,672)	70,426 (4,672)
$^{\#}R_{split}$ (%)	25.7 (73.0)	18.8 (54.5)	18.4 (52.1)
$^{\#}$CC*	0.983 (0.800)	0.991 (0.896)	0.991 (0.897)
$^{\#}$I / σ(I)	3.6 (1.5)	3.9 (1.7)	3.9 (1.8)
$^{\#}$Completeness (%)	100 (100)	100 (100)	100 (100)
$^{\#}$Multiplicity	109 (78)	145 (105)	153 (110)

($^{\#}$) statistics refer to the subset of indexed images

($^{\$}$) all data sets were processed using the same unit cell.

Supplementary Table S3: Statistics of pump-power titration SFX data processed during the LT59 beamtime in the assumed space group $P2_12_12_1$

Dataset	Dark	Δt_900 ps	Δt_900 ps	Δt_900 ps
Energy (μJ)	-	3.7	7.5	11
Data collection and processing				
Space group	$P2_12_12_1$	$P2_12_12_1$	$P2_12_12_1$	$P2_12_12_1$
$Unit cell parameters<br>a (Å)<br>b (Å)<br>c (Å)<br>α,β,γ (°)	 <br>60.2 ± 0.1<br>61.6 ± 0.2<br>183.6 ± 0.5<br>90.0	 <br>60.2 ± 0.1<br>61.6 ± 0.2<br>183.6 ± 0.5<br>90.0	 <br>60.2 ± 0.1<br>61.6 ± 0.2<br>183.6 ± 0.5<br>90.0	 <br>60.2 ± 0.1<br>61.6 ± 0.2<br>183.6 ± 0.5<br>90.0
Hits	172,376	135,930	142,757	226,977
Indexed images	18,430	15,574	12,796	19,151
Indexing rate (%)	10.69	11.46	8.96	8,44
Resolution (Å)	25– 1.80 (1.84 – 1.80)	25– 1.90 (1.95 – 1.90)	25– 1.90 (1.95 – 1.90)	25– 1.90 (1.95 – 1.90)
Observations	9,566,432 (417,017)	6,954,961 (321,119)	6,818,329 (318,313)	10,334,563 (483,927)
Unique reflections	66,176 (4, 349)	56,302 (3,688)	56,301 (3,688)	56,302 (3,688)
Rsplit# (%)	23.6 (113.9)	23.6 (82.4)	27.9 (93.4)	22.4 (71.5)
CC*	0.986 (0.774)	0.984 (0.857)	0.975 (0.850)	0.983 (0.900)
I / σ(I)	4.0 (1.0)	4.1 (1.4)	3.8 (1.2)	4.7 (1.5)
Completeness (%)	100 (100)	100 (100)	100 (100)	100 (100)
Multiplicity	168 (96)	120 (87)	139 (86)	210 (131)
Refinement				
Resolution (Å)	18 – 1.8 (1.83 – 1.80)			
Number of reflections	63,917 (2,642)			
R_{free}	25.9 (33.0)			
R_{work}	22.2 (31.1)			

Number of protein atoms	4,297			
Number of ligand atoms	80			
Number of water atoms	235			
B-factor protein (Å^2)	21.96			
r.m.s.d. bond lengths (Å)	0.006			
r.m.s.d. angles (°)	0.813			
Ramachandran favored	96.23			
Ramachandran allowed	3.59			
Ramachandran outliers	0.18			
Rotamer outliers	2.24			
C-beta outliers	0			
Clashscore	2.87			

($) All data sets were processed using the same unit cell.

Supplementary Table S4: Statistics of refinement against extrapolated structure factors at 20 ps, 900 ps, 300 ns, 2 μs including fatty acid (20 ps) and alkane (900 ps, 300 ns, 2 μs) and a water molecule (at 20 ps, 900 ps, 300 ns, 2 μs) in the active site.

Dataset	Δt_20ps_model_fatty_acid	Δt_900ps_model_alkane	Δt_300ns_model_alkane	Δt_2μs_model_alkane
PDB ID entry	7R33	7R34	7R35	7R36
Pump energy (μJ)	11	11	11	11
Refinement strategy	Difference refinement	Difference refinement	Difference refinement	Difference refinement
Resolution (Å)	10– 2.00 (2.02– 2.00)	10– 2.00 (2.02– 2.00)	10– 2.00 (2.02– 2.00)	10– 2.20 (2.23 – 2.20)
Number of reflections	89,568 (2,736)	89,564 (2,749)	89,565 (2,754)	67,248 (2,607)
R_{free}	32.01 (44.91)	37.27 (49.62)	40.94 (48.25)	37.90 (48.63)
R_{work}	26.46 (40.16)	30.63 (42.37)	35.19 (42.78)	29.72 (41.48)
Number of protein atoms	8,630	8,630	8,630	8,630
Number of ligand atoms	160	160	160	160
Number of water atoms	392	392	392	392
B-factor protein (Å^2)	34.39	34.59	31.98	33.48
r.m.s.d. bond lengths (Å)	0.008	0.008	0.002	0.009
r.m.s.d. angles (°)	1.025	1.026	0.451	1.070
Ramachandran favored	94.70	93.18	94.08	92.91
Ramachandran allowed	4.49	6.01	5.21	5.75
Ramachandran outliers	0.81	0.81	0.72	1.35
Rotamer outliers	5.55	5.99	2.44	7.44
C-beta outliers	0	0	0	0
Clashscore	6.57	9.26	5.54	11.83

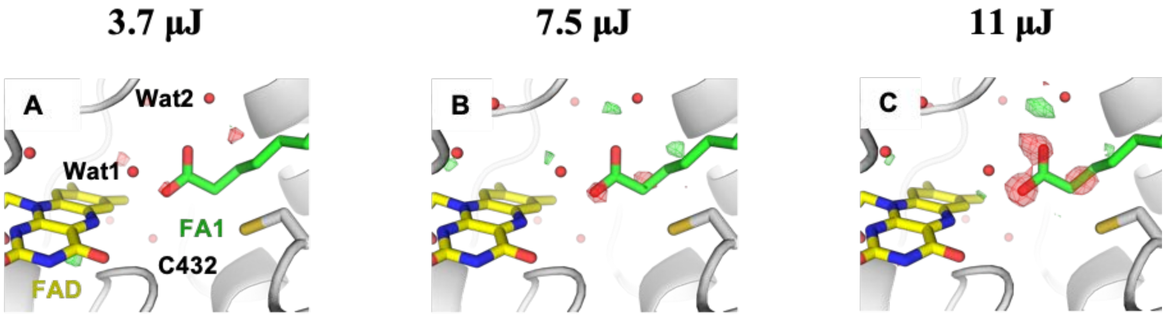

Supplementary Figure S1: **Fourier difference maps at 900 ps at three different pump-pulse energies using data processed in the** ***P2₁2₁2₁*** **space group**

q-weighted Fourier difference electron density maps calculated between SFX light (Δt = 900 ps) data sets at different pump-laser energies and the dark data set ($F_{obs}^{light\_900ps\_E}$ - F_{obs}^{dark}); with E = 3.7 µJ (A), 7.5 µJ (B), and 11 µJ (C) at 1.9 Å resolution. Maps are contoured at +3.5 r.m.s.d. (green) and -3.5 r.m.s.d. (red). The cryo MX dark-state model (PDB entry code 6YRU) is overlaid in panels A-C with FAD in yellow, the fatty acid substrate (FA1) in green and the protein in light grey. The maps were calculated with 18 430 dark images, and with 15 574, 12 796 and 19 151 light images in panels A-C, respectively.

A

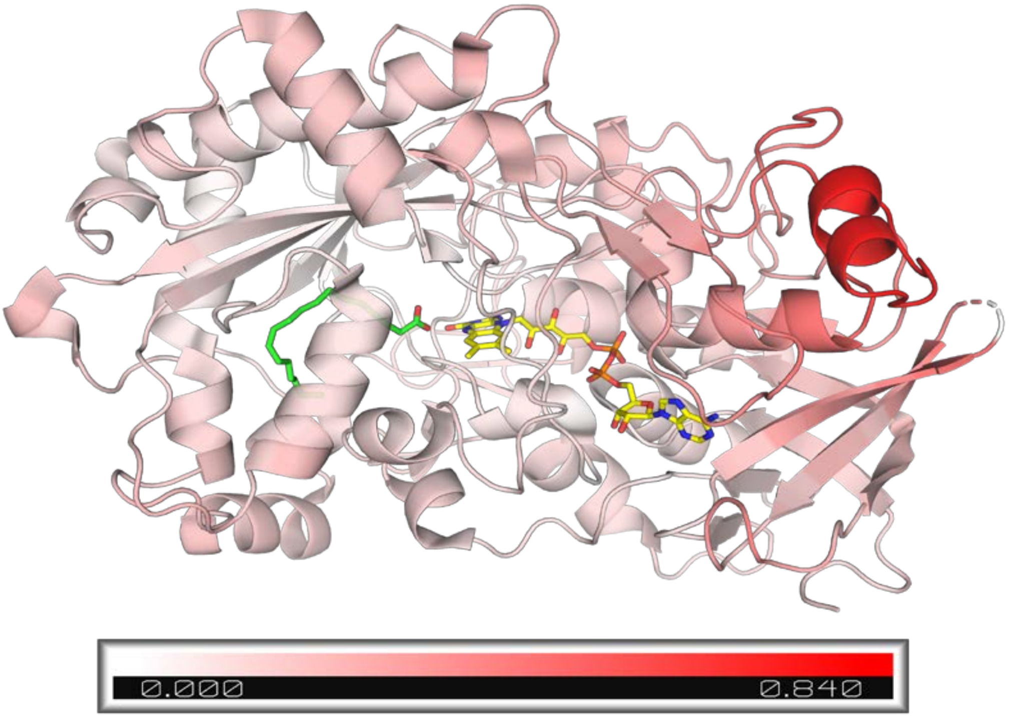

B

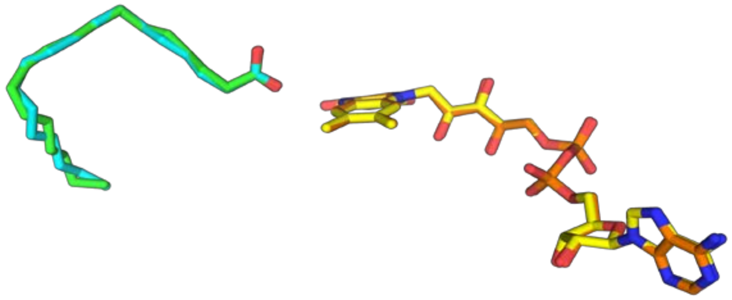

Supplementary Figure S2: Conformational differences between monomers A and B in the asymmetric unit

(A) Cartoon model of monomer A, colored according to the distance between equivalent $C\alpha$ atoms in monomers A and B ($distance = \sqrt{(x_2 - x_1)^2 + (y_2 - y_1)^2 + (z_2 - z_1)^2}$). The color code ranges from white (smaller distances) to red (larger distances; maximum is 0.84 Å) and reflects the distance between equivalent $C\alpha$ atoms in monomers A and B. Carbon atoms of the fatty acid and the FAD of monomer A are shown as green and yellow sticks, respectively. (B) Overlay of FAD/substrate of molecule A and B shown in yellow/green and orange/cyan, respectively.

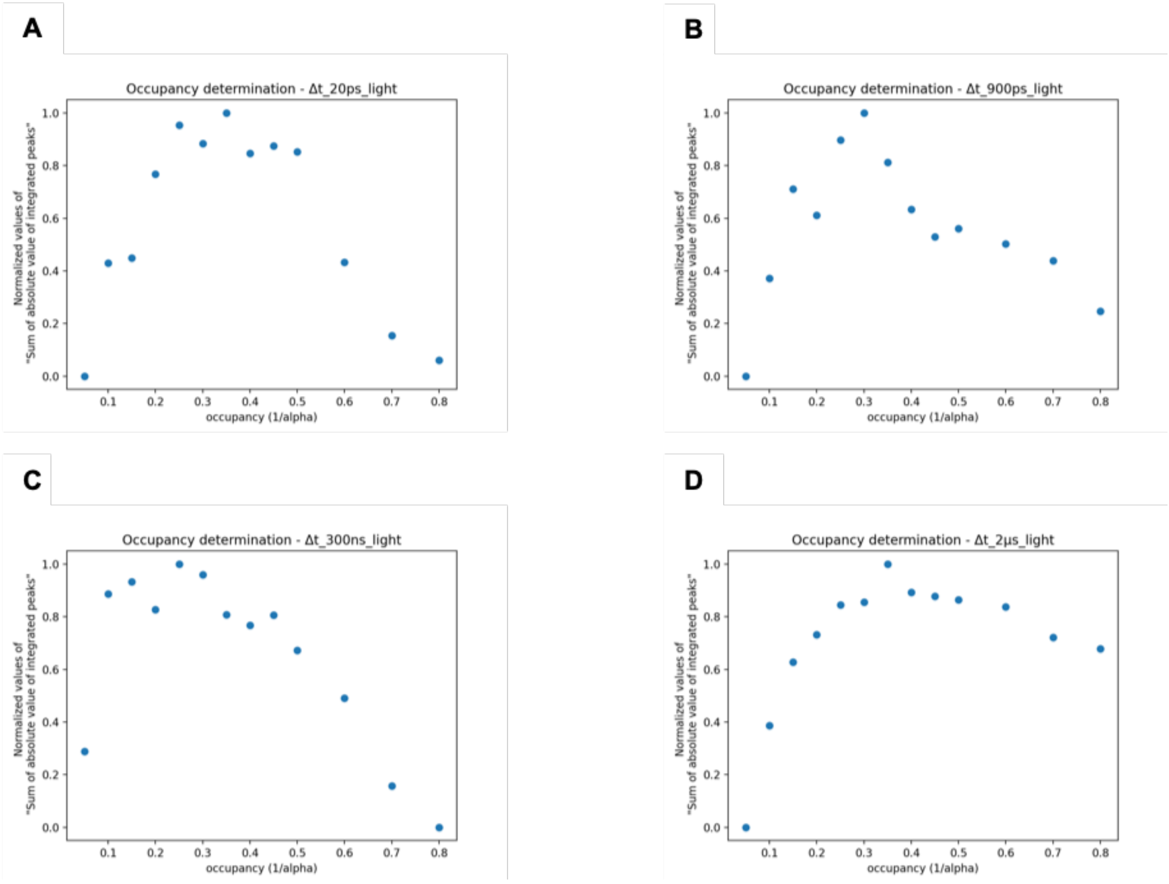

Supplementary Figure S3: Occupancy determination at the four-time delays

Determination of the occupancy, corresponding to the inverse of the weighting factor α of the Δt_light structures with Δ*t* = 20 ps (*A*), 900 ps (*B*), 300 ns (*C*,) at 2 Å resolution and Δ*t* = 2 μs (*D*) at 2.2 Å resolution. The ratio of integrated peaks in the $mF_{ext}^{\Delta t\_300ns} - DF_{calc}^{dark}$ map and of integrated peaks in the $F_{obs}^{\Delta t\_light} - F_{obs}^{dark}$ maps, normalized to its maximum value, is plotted as a function of the occupancy. Only peaks around the fatty acid in the active site were used for α determination.

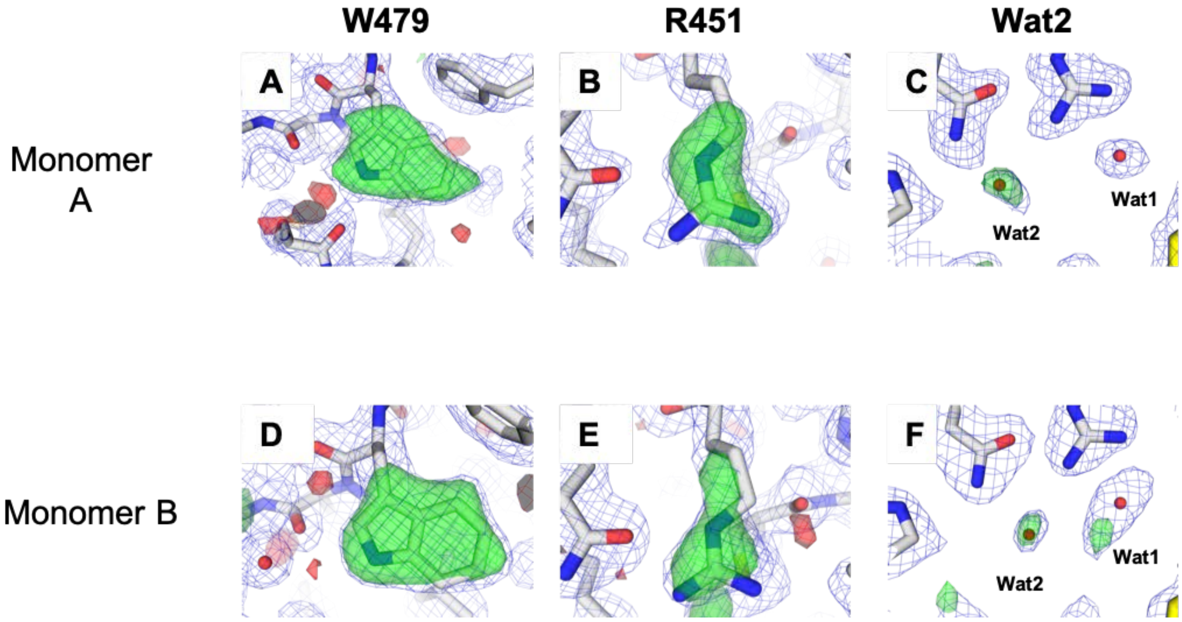

Supplementary Figure S4: Extrapolated electron density maps at 300 ns after omitting Wat2, and the side chains of W479 and R451

Extrapolated electron density maps $2mF_{ext}^{\Delta t\_300ns} - DF_{calc}$ (1 r.m.s.d., blue) and $mqF_{ext}^{\Delta t\_300ns} - DF_{calc}$ maps (+3 r.m.s.d., green; –3 r.m.s.d., red), calculated between the dark and the light data set at 300 ns and phased with a refined model from which the side chains of W479 (A, D) or R451 (B, E), or Wat2 (C, F) have been omitted. The dark state model (PDB entry code 6ZH7) is overlaid. Monomers A and B are shown in panels A, B, C and D, E, F respectively.

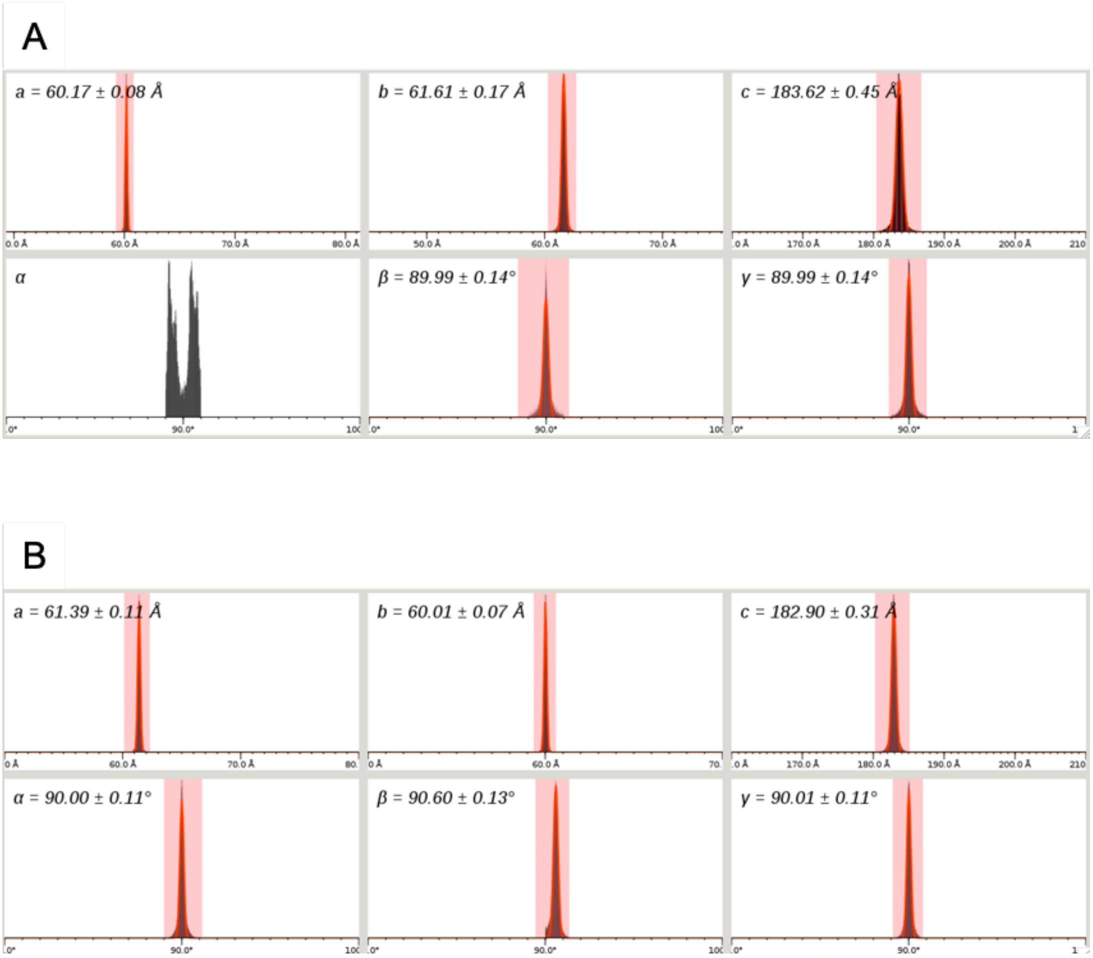

Supplementary Figure S5: Screenshot of cell_explorer from CrystFEL showing the unit cell parameter distributions for dark datasets resulting from processing with $P2_12_12_1$ (A) and $P2_1$ (B)

All unit cell parameters could be well fitted according to a Gaussian distribution (red line) except for the α angle of the $P2_12_12_1$ data (A) that featured a double peak centered at 89.3° and 90.5°.

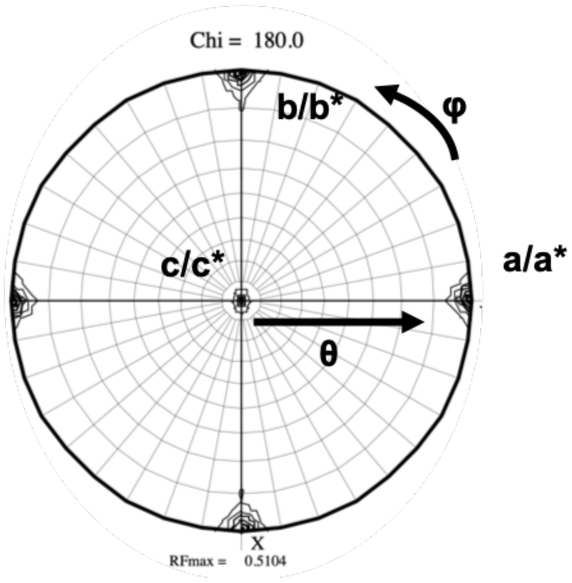

Supplementary Figure S6: **Stereographic projection of the self-rotation function of the dark dataset (PDB code 6ZH7)**

The self-rotation function of the FAP crystal was calculated using data in the 10 – 2.5 Å resolution range and the 2-fold (χ = 180°) section was plotted above a threshold of 25 σ with increments of 0.9 σ. The crystallographic axes are c/c* along z (center of the plot), a/a* along x (θ=90, φ=0), b/b* along y (θ=90, φ=90). The crystallographic axis of $P2_1$ along b/b* is evident, as well as are peaks of similar height along a/a* that stem from NCS linking FAP molecules A and B within the asymmetric unit.

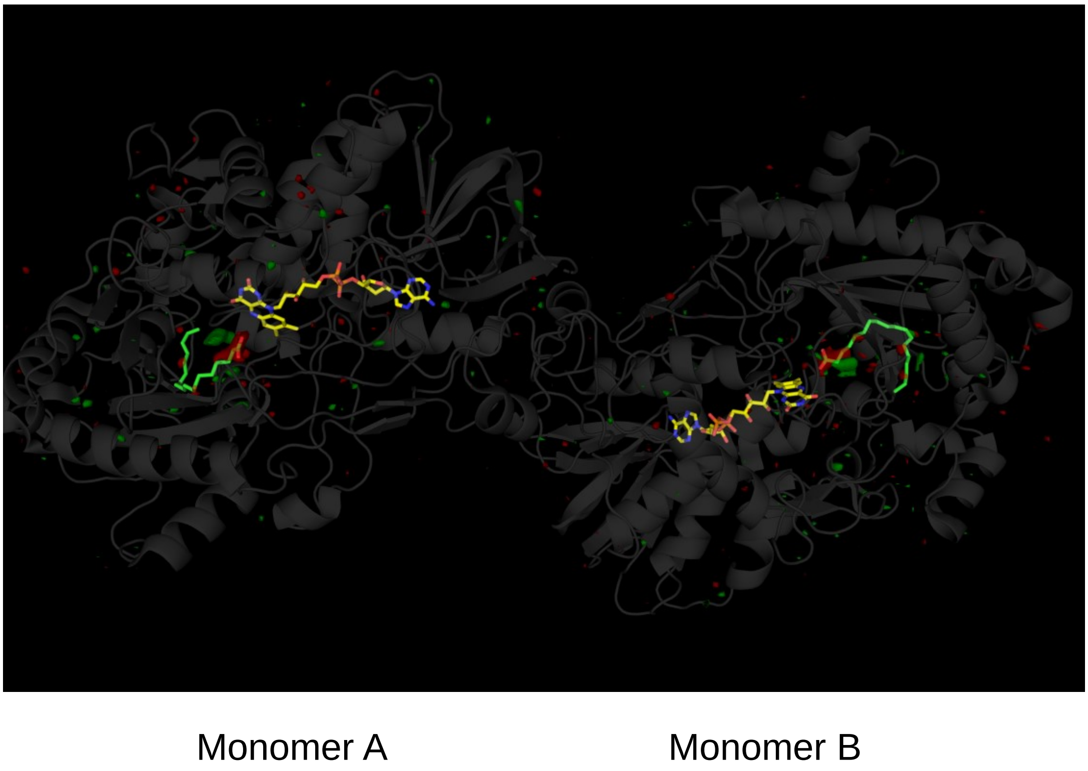

Supplementary Figure S7: Fourier difference map at 300 ns in the asymmetric unit

q-weighted Fourier difference electron density map calculated between the light and dark data sets $F_{obs}^{\Delta t\_300ns} - F_{obs}^{dark}$ at 2 Å resolution. The map is shown at +3.5 r.m.s.d. (green) and -3.5 r.m.s.d. (red). FAD and the C18 fatty acid substrate are shown in yellow and green, respectively. The protein of monomer A is shown in grey.

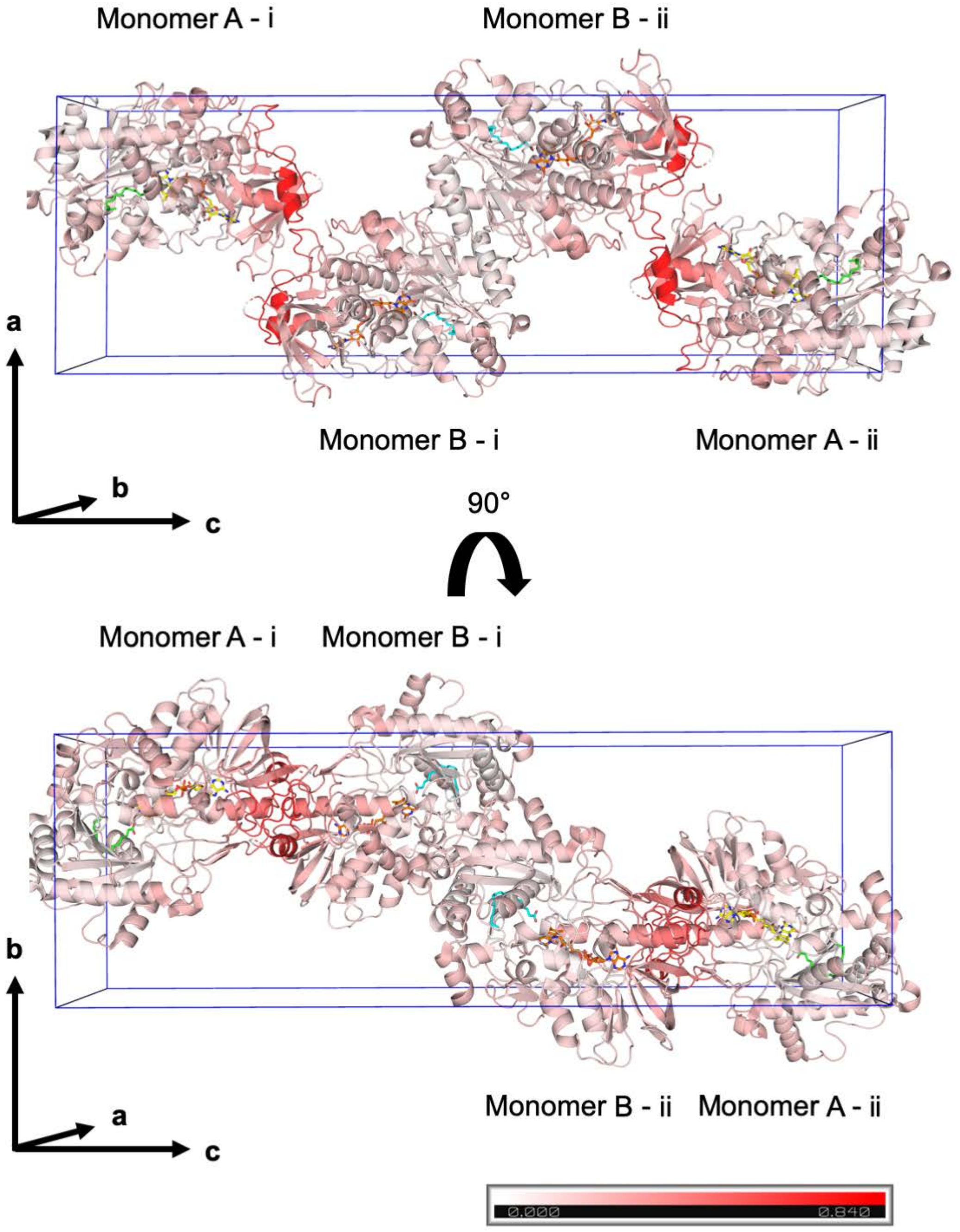

Supplementary Figure S8: Unit cell content of dark-state *Cv*FAP in the P2_1 space group (PDB entry code 6ZH7)

Monomers A and B in the asymmetric units i and ii are shown. The monomers are colored according to the distance between equivalent *Cα* atoms in monomers A and B as in Supplementary Figure S2.

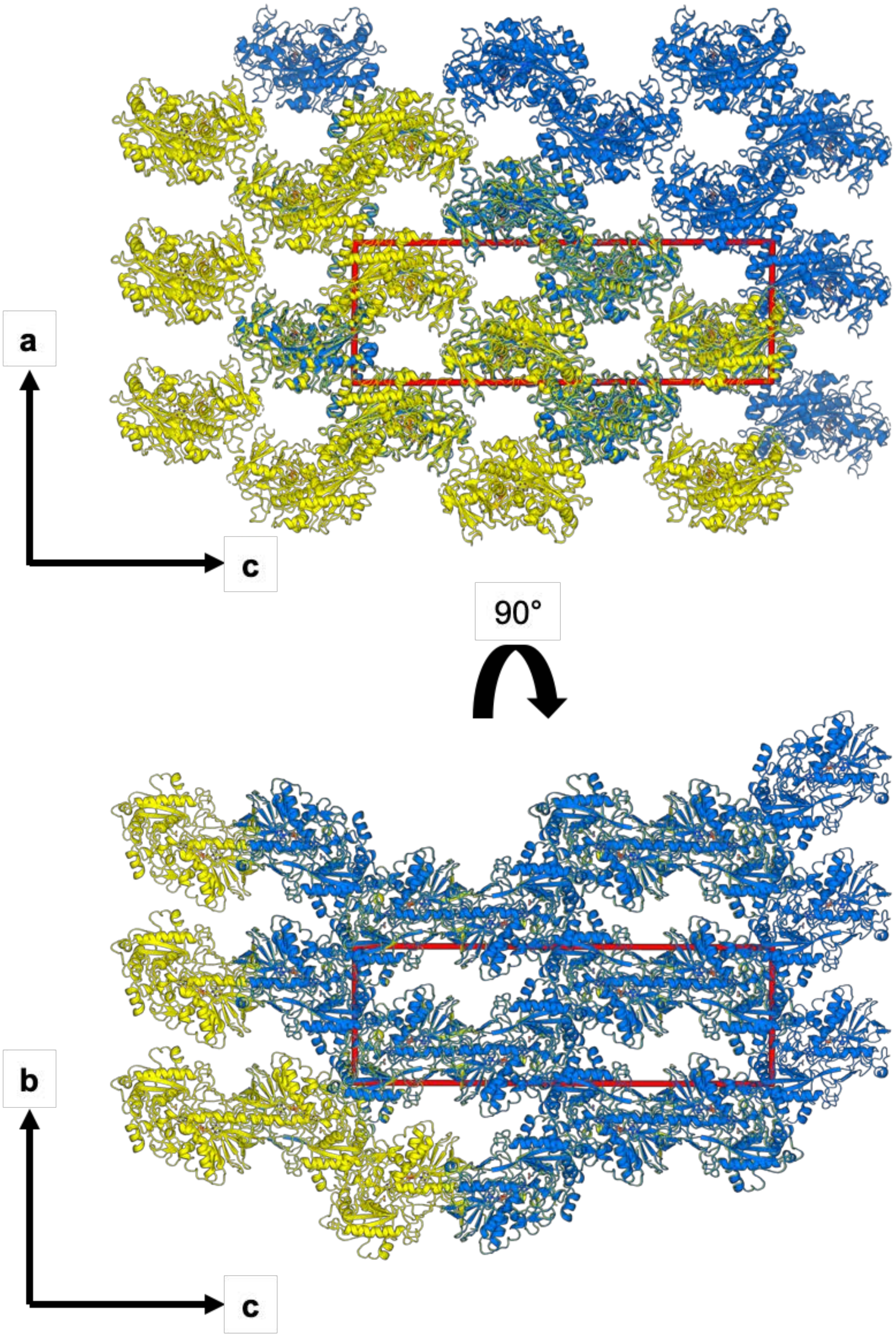

Supplementary Figure S9: Crystal packing in $P2_1$ and $P2_12_12_1$ space groups

The crystal packings of dark-state *Cv*FAP in the $P2_1$ (yellow) and the $P2_12_12_1$ (blue) space groups are shown. The unit cells are shown in red for both space groups: a = 61.4 Å, b = 60.0 Å, c = 182.9 Å and α = 90.0°, β = 90.6°, γ = 90.0° for $P2_1$ (Supplementary Table S1) and a = 60.2 Å, b = 61.6 Å, c = 183.6 Å and α, β, γ = 90.0° for $P2_12_12_1$ (Supplementary Table S3). The superposition indicates that the $P2_1$ packing is only a minor deviation from the $P2_12_12_1$ packing.
